# King Edward's Hospital Fund for London: The Distribution Committee's Report

**Published:** 1915-12-25

**Authors:** 


### The Distribution Committee's Report.

(1) The Governors and General Council have this year fixed the sum available for distribution amongst the London hospitals at £133,500, being the same amount as in 1914.

(2) The Distribution Committee have continued to encourage the hospitals in the policy of deferring all schemes of capital expenditure that are not of exceptional urgency. In a few cases grants are recommended in support of schemes of this character sanctioned during the year, while further assistance from the Fund has in some instances been required in aid of schemes which were already in hand before the outbreak of war. The result is that the total amount of the grants towards buildings and improvements is rather larger than in 1914, amounting to £15,975, as against £12,350 last year. The total amount allotted to maintenance is correspondingly reduced from £121,150 to £117,525, but it still exceeds the total given for maintenance in 1913.

(3) Although the awards are made in respect of the work of the current year and are based upon a consideration of all the known circumstances of each institution, the evidence as to financial position is, as usual, derived primarily from the last audited and published income and expenditure accounts and balance-sheets—namely, those to December 31, 1914. As the circumstances of the different hospitals often vary from year to year, it must not be assumed that a reduction in the grant to any particular institution, whether for maintenance or for capital purposes, or even the absence of a grant, implies dissatisfaction.

### The Effects of the War.

(4) As was explained in last year's Report, the effects of the first few months of the war on the statistics and finances of the hospitals, as well as the consequences of placing beds during that period at the disposal of the naval and military authorities, were reflected in the accounts of the various institutions for the year 1914. These points have received the fullest consideration in making the awards of the present distribution. The effects of similar causes during 1915, in the course of which a much larger number of beds have been provided for sick or wounded sailors and soldiers for much longer periods, will be reflected in this year's accounts. These will also show the effect of the increases in the cost of working and the very large demands which have been made upon the purses of the public in other directions during the same period. The results of these factors during 1915 could only be very roughly estimated at the present time, and will not come fully before the Distribution Committee until they have to consider their next distribution.

(5) The number of hospitals applying for grants is 104, being the same number as last year.

(6) In support of the amalgamation of the Hospital for Diseases of the Throat, Golden Square, and the London Throat Hospital, Great Portland Street, reported last year, a second instalment is payable this year of the maintenance grant of £900 a year subject to the usual annual inspection, which was promised for five years, together with a capital grant of £10,000. Out of the moneys set aside or promised towards a general scheme of amalgamation of the throat, nose, and ear hospitals, there still remain unallocated a capital sum of £10,500, and maintenance grants not exceeding £1,100 a year. The General Council at its meeting on December 16, 1914, gave the Distribution Committee power to draw upon the balance of the capital and maintenance grants so promised or set aside, in such proportions as they may consider appropriate, in the event of any further amalgamation scheme being brought to a satisfactory conclusion at a time when a meeting of the Governors and General Council is not pending. No action has been called for during the current year.

(7) As a consequence of the postponement, during the war, of the less urgent schemes of capital expenditure, the following grants, made in 1913 and retained by the Fund until required, will lapse at the end of the present year unless renewed by the Council, viz.:—Queen Charlotte's Lying-in Hospital, £1,000 to new out-patients' department. St. Mary's Hospital for Women and Children, Plaistow, £1,500 to new nurses' home and out-patients' department. The grant of £2,000 towards the new nurses' home now in course of erection at the West London Hospital has also not yet been applied for. The Committee recommend that all these grants be renewed.

### Economy of Management.

(8) The Distribution Committee have continued to take into consideration economy of management as well as efficiency. They recognise that under the conditions produced by the war there are many factors tending inevitably to raise the cost of working. They have, therefore, directed their attention specially to those instances where the relative cost at a particular hospital, as compared with the average cost at similar institutions, appears to call for remark. By this method any increases due to circumstances common to all hospitals are automatically eliminated, and comment is reserved for cases where the cost has reached an exceptionally high level and therefore seems to require an individual explanation.

(9) The Committee desire again to express their indebtedness to the Fund's visitors for their very valuable reports on the hospitals applying for grants. Many of the inspections have been made at considerable personal sacrifice by visitors, both medical and lay, whose duties have been largely extended by special work connected with the war, and who have, in consequence, had more than usual difficulty in sparing the necessary time to carry out their investigations.

The details of the awards to the various hospitals are appended.

### THE KING'S FUND.

### Grants to Hospitals Recommended by the Distribution Committee.

*The Distribution Committee desire to draw attention to the fact that it must not be assumed that the absence of a grant implies dissatisfaction.*

Alexandra Hospital for Children, £200.

Beckenham Cottage Hospital, £25. Belgrave Hospital, £850. Blackheath and Charlton Hospital, £100. Bolingbroke Hospital, £800. British Hospital for Mothers and Babies, Woolwich, £150, a grant of £1,500 was set aside in 1913 towards the building of the amalgamated hospital, in accordance with scheme to be approved.

Canning Town Women's Settlement Hospital (late Medical Mission), £450. Central London Ophthalmic Hospital, £350. Central London Throat and Ear Hospital; capital grants amounting to £10,500 are set aside and promised for the purpose of an amalgamation of the throat, nose, and ear hospitals (see paragraph 6 of Report). Charing Cross Hospital, £4,000, of which £1,000 to improvements in accordance with the scheme submitted to the Fund. Chelsea Hospital for Women, £2,000, of which £1,500 to rebuilding on new site in accordance with the scheme submitted to the Fund. Cheyne Hospital for Sick and Incurable Children, £100. City of London Hospital for Diseases of the Chest, Victoria Park, £3,000, of which £750 to improvements and £250 to erection of country sanatorium, in accordance with the schemes submitted to the Fund. City of London Lying-in Hospital, £500. Clapham Maternity Hospital, £600, of which £500 to reconstruction in accordance with the scheme submitted to the Fund.

"Dreadnought" Hospital, £3,750

East End Mothers' Lying-in Home, £300. East London Hospital for Children, £2,500. Eltham and Mottingham Cottage Hospital, £50.

Finchley Cottage Hospital, £25. Florence Nightingale Hospital for Gentlewomen, £200. French Hospital, £200.

General Lying-in Hospital, £250. German Hospital, £100. Gordon Hospital for Fistula, etc., £25. Great Northern Central Hospital, £3,750, of which £750 to reduce general fund debt. Grosvenor Hospital for Women, £350. Guy's Hospital, £7,000, of which £1,000 to building improvements in accordance with the scheme submitted to the Fund.

Hampstead General Hospital, £3,000, of which £1,100 to reduce general fund debt, and £150 as agreed upon amalgamation with North-West London Hospital. Consider that greater efforts should be made to obtain local financial support. Hornsey Cottage Hospital, £25. Hospital and Home for Incurable Children, Hampstead, £50, in consideration of the fact that curable cases are admitted. Hospital and Home for Sick Children, Sydenham, £325, of which £75 towards central heating. Hospital for Consumption, Brompton (including Sanatorium at Frimley), £2,600. Hospital for Diseases of the Throat, £900, maintenance grant, as agreed, upon amalgamation with the London Throat Hospital (see paragraph 6 of Report). Hospital for Epilepsy and Paralysis, £1,000. Hospital for Sick Children, £3,000. Hospital for Women, Soho Square, £1,000. Hospital of St. John and St. Elizabeth, £400.

Infants' Hospital, £100. Italian Hospital, £300.

Kensington and Fulham General Hospital, £25. Kensington Dispensary and Children's Hospital, £25. King Edward Memorial Hospital (Ealing), £400. King's College Hospital, £5,000, of which £3,000 to reduction of debt on removal to South London, making £59,000 in all contributed by the Fund to this purpose.

Leyton, Walthamstow, and Wanstead Children's and General Hospital, £400. London Fever Hospital, £100. London Homœopathic Hospital, £1,000, of which £250 to reduce general fund debt. London Hospital, £12,000, of which £2,000 to new nurses' home in accordance with the scheme submitted to the Fund. London Lock Hospital, £2,500, of which £2,000 to maintenance of Harrow Road Hospital, and £500 to supplementary cost of reconstruction of Dean Street Hospital. London Temperance Hospital, £1,500.

Maternity Charity and District Nurses' Home, Plaistow, £125. Memorial Hospital, Mildmay Park, £100. Metropolitan Hospital, £3,000. Middlesex Hospital, £5,250, of which £1,250 to improvements. The grant is made on condition that the accounts of the hospital for 1915 will show no payments to have been made to or on account of the Medical School out of general funds subscribed for the relief of the sick poor, and that the expenses of the Bland-Sutton Institute of Pathology will be allocated in the accounts for 1915 in accordance with a scheme of apportionment to be approved by the Fund. Middlesex Hospital Cancer Charity, £100. Mildmay Mission Hospital, £600. Miller General Hospital for South-East London, £1,500.

National Hospital for Diseases of the Heart, £100. National Hospital for the Paralysed and Epileptic, £2,750. Nelson Hospital (late South Wimbledon, etc.), £350, of which £50 to fire escape staircase from children's ward, and £50 in improvements in nurses' accommodation in accordance with the scheme submitted to the Fund. New Hospital for Women, £750. Norwood Cottage Hospital, £150, of which £100 to recent improvements.

Paddington Green Children's Hospital, £1,000, of which £400 to reduce debt. Passmore Edwards Acton Cottage Hospital, £75. Passmore Edwards East Ham Hospital, £75. Passmore Edwards Hospital for Willesden, £25. Passmore Edwards Cottage Hospital, Wood Green, £50. Poplar Hospital, £250. Prince of Wales's General Hospital, £5,000, of which £2,000 to reduce debt.

Queen Charlotte's Lying-in Hospital, £750. Queen's Hospital for Children, £3,000, of which £500 to reduce debt.

Royal Dental Hospital of London, £400. Royal Eye Hospital, £350. Royal Free Hospital, £4,500, of which £2,000 to reduction of debt on rebuilding of out-patient department in accordance with the scheme submitted to the Fund. Royal Hospital for Diseases of the Chest, City Road, £1,000, of which £500 to reduce debt. Royal Hospital, Richmond, £200. Royal London Ophthalmic Hospital, £2,250. Royal National Orthopædic Hospital, £1,750. Royal Waterloo Hospital for Children and Women, £1,750, of which £250 to reduce debt. Royal Westminster Ophthalmic Hospital, £50.

St. Columba's Hospital, £75. St. George's Hospital, £5,000. St. John's Hospital for Diseases of the Skin. St. John's Hospital, Lewisham, £200. St. Luke's House, £25. St. Mark's Hospital, £300. St. Mary's Hospital, £4,000. St. Mary's Hospital for Women and Children, Plaistow, £800. St. Monica's Home Hospital, £100, of which £50 towards erection of fire escape staircase. St. Peter's Hospital for Stone, £350, of which £150 towards erection of fire escape staircase. St. Saviour's Hospital, Osnaburgh Street, £150. Salvation Army Maternity Hospital, £200. Samaritan Free Hospital, £1,250, of which £250 to recent improvements. Santa Claus Home, £25. Stoke Newington Home Hospital for Women, £25.

University College Hospital, £4,250.

Victoria Hospital for Children, £800.

West End Hospital for Diseases of the Nervous System, £250. Western Ophthalmic Hospital, £500, to reduction of debt on rebuilding of out-patient department. West Ham and Eastern General Hospital, £2,500. West London Hospital, £4,000, of which £1,000 to new nurses' home in accordance with scheme submitted to the Fund. Westminster Hospital, £4,050, of which £3,750 to maintenance, and £300 annual grant to acquisition of new site as agreed. Wimbledon Hospital, £100. Woolwich and Plumstead Cottage Hospital, £25.

**Total Grants to Hospitals for the Year 1915, £133,500.**

(*Continued on page* iv.)

